# Acute systemic inflammation effect on cerebrospinal fluid and blood biomarkers of brain inflammation and injury in dementia: a study in acute hip fracture and memory clinic patients‐ the Ascribed programme

**DOI:** 10.1002/alz70856_099501

**Published:** 2025-12-24

**Authors:** Chris Fox, Jane Cross, Henrik Zetterberg, Lee Shepstone, Colm Cunningham, Alasdair MacLullich

**Affiliations:** ^1^ University of Exeter, Exeter, Devon, United Kingdom; ^2^ NIHR Healthtech research centre, Exeter, United Kingdom; ^3^ University of East Anglia, Norwich, United Kingdom; ^4^ UK Dementia Research Institute at UCL, London, United Kingdom; ^5^ UCL Institute of Neurology, London, United Kingdom; ^6^ University College London, London, United Kingdom, London, United Kingdom; ^7^ Department of Psychiatry and Neurochemistry, Institute of Neuroscience and Physiology, the Sahlgrenska Academy, University of Gothenburg, Molndal, Sweden; ^8^ UEA, Norwich, United Kingdom; ^9^ Trinity College Dublin, Dublin, Ireland; ^10^ University of Edinburgh, Edinburgh, United Kingdom

## Abstract

**Background:**

Epidemiological, PET imaging, genome‐wide association and animal research suggests inflammation contributes to Alzheimer's disease (AD) and other dementias, however there is no consensus on its precise contribution. Human and animal studies show acute systemic inflammation, arising from infection, fracture or co‐morbid inflammatory disease, accelerate dementia. ASCRIBED aimed to investigate the relationship between acute hip fracture, inflammation and brain injury in dementia.

**Materials and Methods:**

Participants were recruited with dementia and hip fracture, those with hip fractures but no dementia, and a control group of individuals with dementia but no acute illness or injury. Cerebrospinal fluid (CSF) and blood samples were collected to measure inflammatory markers and brain injury biomarkers. We recruited from 37 UK hospital sites, drawing CSF and blood from participants with dementia and hip fracture (*n* = 191), participants with no dementia and hip fracture (*n* = 218), participants with confusion and hip fracture (*n* = 80) and a Norwegian cohort of community‐dwelling participants with dementia (*n* = 181).

**Results:**

Most participants were women (68%) across the groups. *APOE3/E3* was the commonest genotype in UK samples (mean 56%). Multi‐morbidity as measured by the Charlson index was highest in the dementia fracture group. Despite the stable dementia group showing the expected CSF elevation in phosphoTau (*p* <0.001) and reduction in CSF Ab1‐42 (*p* <0.001) compared to all other groups, the axonal damage marker neurofilament light chain (NFL) was significantly higher in those with hip fracture than in stable dementia and was highest in those with hip fracture and dementia (*p* <0.001). CSF NFL levels were correlated most strongly with blood CSF barrier permeability, as measured by Q_albumin_ (*p* <0.001).

**Conclusions:**

Our findings show hip fracture leads to an increase in axonal injury, significantly beyond that observed in patients with stable dementia. This acute injury appears more severe in those with existing dementia. Additionally, NFL levels were correlated with blood CSF barrier permeability which may suggest a link between axonal damage and BBB disruption. Further research is needed to elucidate the underlying mechanisms of this acute brain injury and explore potential therapeutic interventions.

